# Saliva and Oral Diseases

**DOI:** 10.3390/jcm9020466

**Published:** 2020-02-08

**Authors:** Emanuela Martina, Anna Campanati, Federico Diotallevi, Annamaria Offidani

**Affiliations:** Dermatology Clinic, Department of Clinical and Molecular Sciences, Polytechnic Marche University, via Conca 71, 60126 Ancona, Italy; ema.martina@gmail.com (E.M.); federico.diotallevi@hotmail.it (F.D.); a.offidani@ospedaliriuniti.marche.it (A.O.)

**Keywords:** biomarkers, saliva, oral cancer, oral lichen planus, psoriasis, oral diseases

## Abstract

Saliva is a fascinating biological fluid which has all the features of a perfect diagnostic tool. In fact, its collection is rapid, simple, and noninvasive. Thanks to several transport mechanisms and its intimate contact with crevicular fluid, saliva contains hundreds of proteins deriving from plasma. Advances in analytical techniques have opened a new era—called “salivaomics”—that investigates the salivary proteome, transcriptome, microRNAs, metabolome, and microbiome. In recent years, researchers have tried to find salivary biomarkers for oral and systemic diseases with various protocols and technologies. The review aspires to provide an overall perspective of salivary biomarkers concerning oral diseases such as lichen planus, oral cancer, blistering diseases, and psoriasis. Saliva has proved to be a promising substrate for the early detection of oral diseases and the evaluation of therapeutic response. However, the wide variation in sampling, processing, and measuring of salivary elements still represents a limit for the application in clinical practice.

## 1. Introduction

Saliva is a biological fluid secreted by major and minor salivary glands. The major salivary glands are the parotid, submandibular, and sublingual glands. Minor salivary glands are widely disseminated throughout the entire oral cavity. Saliva provides lubrication; facilitates mastication, digestion, and taste; it has antimicrobial properties; and serves as buffer for acidic food. Moreover, saliva inhibits the demineralization of teeth and protects from caries [1]. The physiological secretion generates 0.75–1.5 L per day, with a decrease during the night [2]. Saliva contains 99% water and proteins for the remaining 1% (mucins, enzymes, immunoglobulins), electrolytes, lipids, and inorganic substances [3].

There are many advantages to employing saliva as a substrate for diagnostic analysis. Its sampling is fast, inexpensive, non-invasive, and well tolerated by children and people with disabilities; moreover, it is a safe procedure for healthcare providers [4]. Many serum substances enter saliva through passive diffusion, active transport, or extracellular ultrafiltration [5]. Obviously, compared with blood, levels of several analytes are lower, which was an obstacle until a few years ago [6]. Nowadays, highly sensitive molecular methods are available and can be used in the detection of many elements in saliva, despite their dimensions and concentrations [7].

In recent decades, enormous progress has been made in early diagnosis and screening for many diseases, especially for neoplastic conditions. However, some of these methods are invasive or expensive, and for certain conditions, accurate tests are still not available. This is the case for oral cancer, the sixth most common cancer worldwide, frequently diagnosed at an advanced stage with a 5 year survival rate of 50% [8].

In accordance with Biomarkers Definitions Working Group 2011, a biomarker is a characteristic that can be objectively measured and evaluated as indicator of normal biological or pathogenic processes, or as an indicator of pharmacologic response to therapeutic interventions [9]. The detection of salivary biomarkers and their use in clinical practice in the near future is one of the most ambitious aims of contemporary researchers.

## 2. Materials and Methods

The review was conducted in accordance with the PRISMA (Preferred Reporting Items for Systematic Reviews and Meta-Analyses) checklist. A search in the PubMed database was carried out using the keywords “saliva”, “salivary”, “biomarkers”, “oral diseases”, “oral lichen planus”, “oral cancer”, and associations between terms. We selected only articles written in English. The papers were selected first by analyzing titles and abstracts, in order to choose a correct match with our topic; full-text articles were then studied and included in the revision.

## 3. Sampling and Processing Techniques

Many factors can alter the composition and total amount of saliva. The time of day, hydration, body position, drugs intake, smoking, psychological stimuli, food assumption, and other factors related to systemic conditions can change the characteristics of saliva in a single subject [10]. A sample of saliva can be collected at rest or after stimulation. This procedure consists of offering a gum or swab to chew, or specific taste stimuli such as citric acid [11]. The stimulation changes not only the volume, but also the composition of saliva; it has been demonstrated that parasympathetic stimulation produces a high flow rate, but sympathetic stimulation produces a small flow richer in proteins and peptides [12]. Consequently, proteome profile and proportion are changeable as a reaction to neural activation [13].

As regards clinical trials, saliva is usually collected at rest (“unstimulated saliva”) after at least 1 h of fasting, without drinking or smoking; the patient must be comfortably seated, avoid oro-facial movements for 5 min, and, just before the sampling, has to rinse their mouth with deionized water [11].

Saliva specimens can be collected from whole saliva or from a single gland (for example, the parotid gland). This procedure, which uses a different method, can be uncomfortable for patients and therefore is rarely used [14]. It should be specified that whole saliva has a higher proportion of non-salivary materials such as food debris, bacteria, desquamated epithelial cells, and leukocytes [15].

The gold standard method is to drain saliva using special devices (Salivette^®^, Sarstedt, Nümbrecht, Germany; Quantisal^®^, Immunalysis, Pomona, CA, USA; Orapette^®^, Trinity Biotech, Dublin, Ireland and SCS^®^ Greiner Bio-One, Kremsmünster, Austria) [16].

Controversies are evident in the literature regarding centrifugation and speed, addition of PIC (protease inhibitor cocktail), and storage temperature. Most authors recommended the use of a protease inhibitor mixture in order to stabilize the substrate; moreover, the samples collected must be immediately stored in ice containers and, after processing, stored at −80 °C [17]. All these steps are necessary for bacterial growth inhibition and the minimal impairment of salivary proteins.

## 4. “Salivaomics”

The term “salivaomics” was coined in 2008 to emphasize the various “omics” found in saliva: genome, transcriptome, proteome, metabolome, and microbiome [18]. Salivaomics has been widely studied in recent years thanks to the advent of more advanced analytical techniques. Nearly 70% of the genome in saliva is human; the remaining 30% belongs to the oral microbiota [19]. The DNA contained in saliva is approximately 24 μg (range 0.2–52 μg), which is almost 10 times lower than in blood, but genotyping techniques require as little as 5 ng/mL of DNA to work effectively [20]. Polymerase chain reaction (PCR) and sequencing arrays can be applied to saliva samples. The analysis of salivary DNA aims especially to detect aberrant DNA methylation, which is the first epigenetic mark of neoplastic alterations [21].

### 4.1. Transcriptomes

mRNA and microRNA secreted from cells can be easily detected in saliva. Reverse transcriptase polymerase chain reaction and microarray are the most commonly used analyses. Zhang et al. first developed a technique to permit stabilization and to process salivary RNA [22]. The great potential of transcriptome study in the early detection of cancer and other diseases has been reported [23,24,25]. More recently, noncoding RNAs (ncRNAs) or microRNAs (miRNA) have been the subject of many studies because of their role in oncogenesis and their great stability in biological fluids, including saliva [26]. MicroRNAs are encoded by genes but are not translated into proteins; it is now generally accepted that these small nucleotides are involved in cell differentiation, proliferation, and survival. Moreover, many studies have already demonstrated the dysregulation of miRNAs in cancer tissues [27,28]. Surprisingly, salivary microRNAs are more stable than mRNAs, which makes this biological fluid a suitable substrate for transcriptome analysis.

### 4.2. Metabolome

The endogenous metabolites are nucleic acids, vitamins, lipids, organic acids, carbohydrates, thiols, and amino acids. The study of the salivary metabolome can provide an overview of the general health status or modification during systemic diseases [29]. In 2010, Sugimoto et al. first used salivary metabolome analysis with capillary electrophoresis and mass spectrometry to detect differences between healthy controls and patients with solid cancer [30]. The authors identified three metabolites that were oral-cancer-specific and eight metabolites that were pancreatic-cancer-specific. Nuclear magnetic resonance (NMR) spectroscopy can detect and measure metabolites in a solution with minimal sample preparation. This quantitative technique is based on the magnetic properties of atomic nuclei [31]. Each compound has a characteristic resonance frequency that makes it easy to distinguish. Moreover, the area under a signal peak is proportional to the concentration of the metabolite [32]. Liquid chromatography–mass spectrometry (LC-MS) is considered the gold standard in metabolomics. In fact, it is able to analyze an enormous range of analytes with a greater sensitivity than NMR [33]. This technique provides very high chromatographic resolution and its results are easily interpretable using libraries of molecular fragmentation patterns [34].

### 4.3. Proteome

The term “proteome” encompasses all proteins in the oral cavity. Saliva contains more than 2000 proteins with a multitude of biological activities [35] and one quarter of salivary proteins are detectable in plasma. The greatest obstacle to salivary proteome analysis is its rapid degradation, which occurs just minutes after sample collection. For this reason, the majority of researchers combine the saliva with protease inhibitor cocktails (PIC) before storage and analysis, as suggested by Xiao et al. [36]. Proteomics takes advantage of NMR spectroscopy and gas and liquid chromatography–mass spectrometry (GC-MS and LC-MS), described above. In this research field, two-dimensional polyacrylamide gel electrophoresis (2D-PAGE) and capillary electrophoresis with electrochemical detection are essential tools [37]. 2D-PAGE, which precedes the advent of 2D-difference gel electrophoresis (2D-DIGE), fractionates proteins on the basis of their isoelectric points in the first dimension and apparent molecular weight in the second [38]. An amphoteric carrier or an ampholyte is added to a gel and subjected to electrophoresis under a continuously regulated temperature. The acrylamide gel is placed in a glass tube and proteins are separated via an isoelectric gradient; it is easy to understand why this method is poorly accurate with multiple samples. 2D-DIGE dramatically improved 2D-PAGE thanks to the possibility of labeling each sample with distinct fluorescent dyes and then reading them using a laser scanner. Immunoassay is one of the most commonly used analytical techniques to detect the expression of an antibody or an antigen in a test sample. Enzyme-linked immunosorbent assay (ELISA) has been used for a variety of applications including diagnostic tools and quality controls [39]. The four basic setups are direct, indirect, sandwich, and competitive ELISAs. Direct ELISA is the simplest format, requiring an antigen and an enzyme-conjugated antibody specific to the antigen [40]. The ELISA method is a sensitive and specific test that rapidly produces results and for these advantages has found a wide field of applications in clinical practice (e.g., in viral serology tests).

### 4.4. Microbiome

The study of microbiota has probably been the largest topic in scientific literature in recent years. In fact, next-generation sequencing has allowed the identification of thousands of phylotypes of microorganism throughout the entire human body, and research is ongoing. About 19,000 microorganisms have been identified in saliva [41]. Oral dysbiosis can lead to periodontal disease [42], caries [43], and some evidence exists supporting an association with cancer and systemic diseases [44,45]. Nowadays, molecular biology methods such as 16S ribosomal RNA (rRNA) gene sequencing, polymerase chain reaction (PCR), and other related PCR-based methods are very popular thanks to their high sensitivity and reproducibility. However, these techniques are no longer employed in routine diagnostics due to their costs. Alternative approaches include electromigration techniques (two-dimensional gel electrophoresis, capillary zone electrophoresis) and MS methods, such as matrix-assisted laser desorption ionization time-of-flight mode (MALDI-TOF MS). MALDI-TOF MS is a fast and accurate method based on the ionization of intact microorganism cells with short laser pulses and the subsequent acceleration of the particles in a vacuum by way of an electric field. Each microorganism has a specific spectrum profile [46].

Histopathology, in some cases with direct immunofluorescence, remains the gold standard for the diagnosis of oral disease. In fact, it is often necessary to perform a biopsy to confirm the diagnosis of bullous diseases (together with DIF) [47], Sjögren’s syndrome [48], and for all lesions suspected for malignancy [49].

## 5. Fields of Application

In this article, we have summarized the latest findings on the use of saliva as a diagnostic tool in oral inflammatory diseases. In particular, we chose the most epidemiologically relevant conditions or where the oral cavity is a typical location of a systemic disease. In fact, mouth disorders can often precede the onset of systemic symptoms (e.g., in bullous pemphigus), and early diagnosis of oral disease can change the prognosis of these patients. In this scenario, the study of salivary biomarkers is a promising tool for early diagnosis and screening in susceptible populations (e.g., in smokers).

### 5.1. Oral Lichen Planus

Oral lichen planus (OLP) is one of the most common chronic inflammatory condition of the oral mucosa, with 0.5%–2% prevalence in adults and a slight predominance in women [50]. OLP affects oral mucosa symmetrically, with a predilection for oral mucosa. Clinically, it is possible to distinguish different aspects: reticular (the most prevalent form), erythematous, ulcerative or erosive, plaque-like, bullous, or papular [51,52]. The histopathology of OLP is typical, with a prominent lymphocyte infiltrate at the interface of epithelium, acanthosis, and degeneration of the basal cell layer [53]. Direct immunofluorescence (DIF) permits deposition of Immunoglobulin M as colloid bodies and C3 in granular and linear patterns in the basement membrane zone to be detected [54]. Although the exact pathogenesis of OLP is mostly unknown, it is believed that autoreactive T cells play a crucial role in the disease. Several risks/triggers factors have been described, such as stress, HCV and viral infections, and drugs [55]. OLP has been classified as a premalignant lesion for its risk of malignant transformation (0.04–1.74 per year) in squamous cell carcinoma (OSCC) [56]. Patients affected by OLP suffer from burning and itching sensations up to a severe pain in the erosive form; the disease has a huge negative impact on quality of life due to impairment in daily activities such as eating or oral hygiene [57]. Published articles focused on salivary biomarkers in OLP are quite recent and concern the diagnosis of OLP, but in particular the early detection of malignant transformation.

In 2018, Sineepat et al. enrolled five OLP patients and five healthy controls using a proteomic approach on saliva with two-dimensional gel electrophoresis followed by mass spectrometry. The authors detected three proteins that showed a potential role in OLP patients (cystatin SA, chain C of human complement component C3c, and chain B of fibrinogen fragment D) and tested with ELISA. All the analytical techniques confirmed with statistical significance that fibrinogen fragment D and complement component C3c were increased and cystatin SA was decreased in OLP patients compared with healthy subjects [58]. Fibrinogen fragments D and C3c play a central role in inflammation, whereas cystatin SA belongs to the cystatin superfamily, a group of cysteine protease inhibitors with antimicrobial activity. In fact, fibrinogen expression and C3 deposition are typical findings in OLP using IFD [54].

A different and more complex panel of proteins was reported by another study, published in 2017 [59]. The study was conducted on 10 patients, investigating with mass spectrometry 108 proteins differentially expressed in OLP subjects in comparison to healthy controls. The first finding was the absence of proteins essential to lubrication and viscoelasticity, supporting the xerostomia symptom frequently reported by patients. The authors interestingly tried to link protein expression in saliva with histological findings in OLP, discussing the known functions of each peptide. In particular, S100A8 and S100A9 (also called MRP8 and MRP14) are calcium- and zinc-binding proteins with a role in inflammation and cytokine production via IL-17. S100A8 can also induce apoptosis via attraction to skin of CD8^+^ cells and natural killer (NK) cells [60]. Another player in the scene of T-cell proliferation and differentiation is AZGP1 (zinc-alpha-2-glycoprotein), which is an adipocytokine [61]. The study also confirmed the crucial role of oxidative stress in OLP; reactive oxygen species (ROS) induce apoptosis and dysfunction in keratinocytes and, moreover, ROS can be further produced from TCD4^+^ lymphocytes infiltrating in OLP in a vicious circle.

Oxidative stress in OLP was previously discussed in 2016 in a case–control study enrolling 62 patients and 30 healthy individuals [62]. The authors demonstrated significant differences between patients and the control group concerning the average concentration of total antioxidant capacity (TAC, determined using the Benzie and Strain method [26]), glutathione (GSH, measured spectrophotometrically), and thiobarbituric-acid-reactive substances (TBARS, determined using the Aust method), which are a product of lipid peroxidation [63]. In patients suffering from OLP, as expected, TAC and GSH had lower values, while TBARS was higher than in healthy controls. More interestingly, patients with an erosive form of lichen had more marked values, demonstrating severe oxidative stress and a great concordance with clinical features. These findings could support the oral or topical use of antioxidants [64].

Many authors have suggested salivary cortisol as a biomarker in OLP [65,66,67,68,69,70]. Cortisol is considered a biological marker of stress and anxiety, the variation of which can alter cytokine profiles [71]. OLP has a double connection with stress: anxiety and stressful events are considered a trigger for OLP onset but, at the same time, oral lichen itself represent a source of stress for patients. In this intricate scenario, the evaluation of salivary cortisol seems to mimic the ancestral question “Which came first, the chicken or the egg?” In fact, data from the literature are controversial, and cortisol is probably not suitable as a biomarker in OLP. As previously discussed, OLP is a T-cell-driven disease; however, it is still unclear if the inflammation is due to Th1 or Th2 expression. In fact, in OLP, there are numerous cytokines expressed both from recruited lymphocytes and from affected keratinocytes, in a mechanism of self-amplification [72]. The evaluation of specific interleukin in saliva is certainly a good trace to detect biomarkers in OLP and, moreover, to design tailored therapies. Nowadays, more consistent results concern IL-6 and IL-8. Interleukin-6 is involved in B- and T-cell differentiation and is able to inactivate p53 with tumor progression of some cancers [73]. Mozaffari et al. revealed in a meta-analysis that IL-6 levels in saliva and serum of OLP patients were significantly higher than in healthy controls, with higher values in saliva than in serum [74]. For this reason, saliva seemed to be more useful than serum for the detection of IL-6. Interleukin-8 is an important mediator of host response to injury and inflammation; it can activate neutrophils, basophils, and T cells [75]. The group of Mozaffari conducted a meta-analysis on this topic [76]. The most interesting finding was that IL-8 plays a key role in the transformation from reticular to erosive form of lichen, probably due to the loss of efficacy in the repairing mechanisms of keratinocytes [77]. IL-8 also revealed a potential application in therapeutic monitoring, as demonstrated via its decrease in saliva after dexamethasone administration [78].

### 5.2. Oral Cancer

Oral cancer is the sixth most common cancer worldwide [79] with a higher incidence in India, because of the chewing of areca nut/betel quid [80]. The mortality rate after 5 years from diagnosis is still 50% [79]. Well-known risk factors include tobacco consumption, alcohol abuse, and human papilloma virus infections [81]. The onset of an oral cancer is frequently asymptomatic, but most oral carcinomas develop from premalignant conditions such as leukoplakia and oral lichen planus [82,83]. Nowadays, the gold standard for diagnosis is tissue biopsy, an invasive technique that requires specific training and creates public health costs [84]. It is therefore easy to understand the need for an early detection method for pre-cancer and cancer by validating salivary biomarkers. Above, we discussed the great diagnostic potential of miRNA both in saliva and serum. In 2018, a well-designed study enrolled 30 patients with OLP, 15 patients with OSCC and 15 healthy donors [85]. Saliva samples were analyzed by quantitative RT-PCR for miR-21, miR-125a, miR31, and miR200a. Results showed that miRNA-21 and -125a were, respectively, higher and lower in OSCC patients and in OLP with dysplasia compared to healthy controls with statistical significance. miR-21 has been widely studied in oral, head, and neck cancer and has been postulated that it might have a role in inhibition of tumor suppression and apoptosis [86]. In contrast, miR-125a may act as a tumor suppressor, downregulating target oncogens [87]. Based on these data, the authors suggested a negative prognostic role of decreased salivary miR-125a levels in association with increased salivary miR-21 levels in OLP patients. Ishikawa et al. recently suggested a metabolomics approach to distinguish OLP from OSCC [88]; the authors detected higher levels of 12 salivary metabolites in OSCC patients compared with OLP patients. More specifically, the combination of indole-3-acetate and ethanolamine phosphate showed the best statistical accuracy. The aim of Mikkonen’s research was to investigate the potential of nuclear magnetic resonance (NMR) spectroscopy for detecting the salivary metabolic changes associated with head and neck squamous cell carcinoma (HNSCC). The Authors found two metabolites, fucose and 1,2-propanediol, to be significantly upregulated, whereas proline was significantly downregulated in patients affected by HNSCC. The combination of four salivary metabolites (fucose, glycine, methanol, and proline) together provided maximum discrimination among HNSCC patients and healthy controls [31]. The role of fucosylation of glycoproteins in the development of cancer has been studied in recent years [89]. Ample evidence exists to prove that in normal tissues, fucosylation levels are relatively low, but this rapidly increases during carcinogenesis [90]. Aberrant glycosylation in cancer development is also an investigation area in oral diseases; in particular, researchers have focused attention on sialic acid (N-acetyl neuraminic acid), which is an important terminal sugar in cell membrane glycoproteins and glycolipids. Previous studies have shown elevated levels of salivary sialic acid in various carcinomas, including oral pre-cancer and OC [91,92,93].

The fascinating study of the microbiome has a wide field of application in the oral cavity. An extensive work has just been published regarding the alterations of salivary microbial community in oropharyngeal and hypopharyngeal carcinoma patients. In fact, the microbiome is considered a potential modulator of cancer metabolism [94]. The authors found 13 phylotypes of microorganism as potential diagnostic biomarkers in oral cancer. The role of the microbiome in malignant change in the oral cavity is still controversial because of the lack of large cohort studies. Healy et al. considered the implication of risk factors such as smoking or alcohol consumption in promoting epithelial dysplasia and production of carcinogenic agents [95]. Acetaldehyde (ACH) and N-nitrosamine compounds are potential genotoxic agents that are increased in the saliva of smokers; these compounds can be produced in vitro by microbial cultures [96,97]. In vitro studies have demonstrated the leading role of *Neisseria* species and *Candida* species in ACH production [98,99]. However, one study revealed the reduction of *Neisseria* species in the oral cavity of smokers, with a theoretical improvement of ACH levels [100]. Current theories hypothesize that the presence of these organisms could accelerate the progression of dysplasia towards OSCC in association with predisposing factors such as diet, age, or smoking/alcohol consumption habits in a multifactorial vision.

### 5.3. Blistering Diseases

Bullous pemphigoid (BP) and pemphigus vulgaris (PV) are acquired bullous diseases affecting the mucosa and/or skin. In both diseases, autoantibodies react with adhesion cell mechanisms or with the basement layer, resulting in blistering. Blisters are intraepithelial/intraepidermal in PV, whereas in BP they are subepithelial/subepidermal [101]. The diagnosis is first clinical, then confirmed with histopathology and direct immunofluorescence (IFD). In BP, bullae involving the skin and oral lesions are rare; in contrast, PV frequently begins with oral blistering or oral lesions following cutaneous involvement. IFD reveals IgG and C3 (BP180) deposition on the basement membrane in BP, while in PV it shows intercellular IgG antibody deposition to desmoglein (Dsg) 1 and/or desmoglein 3, which are trans-membrane desmosomal proteins [102]. In recent years, the use of ELISA to detect autoantibodies in the serum of BP and PV patients has entered clinical practice for diagnosis and therapeutic monitoring [101]. Starting from this technique, some authors have proposed the use of saliva as substrate for the research of BP180 and Dsg1 and 3. In 2006, Andreadis et al. first applied ELISA in both the serum and saliva of PV and BP patients, finding a great concordance in serum and saliva levels of Dsg1 and 3, while the BP180 determination on saliva failed [103]. Similar results emerged from Ali’s study [104] on Dsg1 and 3. The potential of salivary testing in PV prognosis and mucosal severity has been investigated in two studies. Hallaji et al. included 50 patients with histologically confirmed PV and performed ELISA for Dsg1 and 3 on serum and saliva samples [105]. There was statistically significant concordance between serum and salivary levels of Dsg; more interestingly, there was a significant relationship between salivary anti-Dsg1 antibody and mucosal severity. The authors explained these data with the loss of integrity in mucosa and the largest transition of antibodies in saliva. The study of De et al. perfectly reproduced this finding and the authors perfectly agreed with the explanation concerning higher Dsg1 levels in severe disease [106]. In contrast to the previously discussed research, one Italian study was designed to assess the use of a BIOCHIP approach compared with ELISA in PV [107]. In fact, the authors considered saliva an unsuitable substrate for autoantibody detection because of the discordance between techniques found when using saliva samples.

### 5.4. Sjögren’s Syndrome

Sjögren’s syndrome (SS) is a systemic autoimmune disease characterized by the inflammation and consecutive destruction of exocrine glands, as well as salivary and lacrimal glands, with the occurrence of a lymphoepithelial sialadenitis [108]. The majority of patients are women of menopausal age; oral manifestations are frequently present at the onset of disease, but some patients develop a systemic disease with the involvement of joints, the gastrointestinal tract, the central nervous system, and with an increased risk of lymphoma [109]. Patients suffering from SS typically complain about xerostomia and its impact on their quality of life [110]. Current research on salivary biomarkers in SS is pursuing a non-invasive diagnostic test, a therapeutic monitoring marker, and, moreover, an early detection of lymphoma onset. One of the current diagnostic approaches is the detection of anti-Ro/SSA and/or anti-La/SSB in serum; studies from different groups have demonstrated the presence of these autoantibodies in the saliva of SS patients [111,112]. The determination of salivary autoantibodies seemed to be effective in discriminating SS patients from patients affected by systemic lupus erythematosus (SLE) [113]. A few studies have investigated cytokine profiles in SS saliva; data from these studies showed significantly higher levels of Th1, Th2, and Th17, in accordance with serum findings [114,115]. The proteomic approach in SS comprises proteins, enzymes, calcium-binding proteins, and immune-related molecules. Summarizing, data from the literature report high levels of inflammatory-phase proteins in saliva that can provide a great indication of gland status [116]. Lee et al. recently published the results of determination of soluble sialic-acid-binding immunoglobulin-like lectin (siglec)-5 in saliva and sera by ELISA [117]. The level of salivary siglec-5 was significantly higher in the saliva from SS patients, which reflects the severity of hyposalivation. Several novel miRNAs have been described in SS [118]. Pauley et al. demonstrated that the expression of miR-146a was significantly increased in SS patients [119]. In Alevizos’ research, another two miRNAs, miR-768-3p and miR-574, were associated with minor salivary gland inflammation in 15 patients with SS [120]. The pathogenesis of autoimmune diseases is a very complex interaction of many factors; epigenetic modifications are now considered crucial to the control of gene expression associated with these diseases [121,122]. Thabet et al. proposed that the dysfunction of salivary gland epithelial cells in SS might be partially linked to epigenetic modifications. Their analysis showed that blood global DNA methylation was reduced in SS patients and the expression of the gene DNMT1, which encodes DNA methyltransferase 1, was decreased compared to healthy controls. In contrast, the expression of the gene Gadd45a, which encodes the growth arrest and DNA-damage-inducible protein GADD45 alpha (GADD45a), was increased [123]. Probably the most interesting field in saliva and SS is the early diagnosis and prevention of MALT-type lymphoma [124]. The neoplasm has an insidious onset, almost asymptomatic, with a fast progression and dissemination. Cui et al. described a triad of markers (anti-cofillin-1, anti-alpha-enolase, and anti-Rho GDP-dissociation inhibitor 2) overexpressed in patients with SS who developed MALT lymphoma compared with SS patients and healthy individuals [125]. Sharma et al. recently examined the role of the microbiome in SS compared to healthy controls [126]. The analysis, performed with DNA isolation and 16rRNA sequencing, revealed four genera (Bifidobacterium, Dialister, Lactobacillus, and Leptotrichia) that were different between the two groups. The results were consistent with previous studies, revealing a role of Actinobacteria and Firmicutes phila [127,128]. More interestingly, Sharma et al. identified a difference in alpha diversity in patients treated with steroids, suggesting the potential role of microbiome analysis in therapeutic response.

### 5.5. Psoriasis

Psoriasis is now classified as an immune-mediated inflammatory disease (IMID) of the skin. It is being recognized that patients with psoriasis are at higher risk of developing systemic co-morbidities, e.g., metabolic syndrome and cardiovascular diseases [129,130].

Oral involvement in course of psoriasis is still debated. Recently, it has been hypothesized that gingivitis and periodontitis share the same underlying inflammatory pathogenic process as psoriasis. Thus, in our previous study, psoriatic patients were investigated for oral mucosa lesion prevalence as well as gum disease. Results displayed an increased association between gingivitis/periodontitis and psoriasis, which may suggest common underlying pathogenic risk factors [131].

Furthermore, salivary secretions, collected from patients with active psoriasis and healthy control subjects, were investigated for expression of interleukin (IL)-1b, IL-6, transforming growth factor (TGF)-β1, IL-8, tumor necrosis factor (TNF)-α, interferon (IFN)-χ, IL-17A, IL-4, IL-10, monocyte chemoattractant protein (MCP)-1, microphage inflammatory protein (MIP)-1a, and MIP-1b using a Multi-Analyte ELISArray Kit (Qiagen, Venlo, the Netherlands). Patients with active psoriasis had significantly higher salivary IL1β, TNF-α, TGF-β, and MCP-1 levels than healthy controls [132].

Thus, saliva can be a valid non-invasive tool for monitoring inflammation in psoriasis [133].

## 6. Conclusions

In the era of precision medicine, salivaomics approaches seem to be a promising field of research. Despite encouraging results reported in this review, there is a large variability in study designs, protocols, sampling collections, and techniques. Moreover, the study of new molecules with new technologies requires a well-established range of values without random decisions. Future studies should standardize accurate methodologies in order to validate new salivary biomarkers in clinical practice.
